# Combined physical activity training versus aerobic activity training in unipolar depressive disorder: a quasi-randomised evaluation study

**DOI:** 10.1007/s40211-023-00464-1

**Published:** 2023-04-21

**Authors:** Andre Berwinkel, Martin Driessen, Thomas Beblo, Matthias Weigelt

**Affiliations:** 1 Universitätsklinik für Psychiatrie und Psychotherapie, Evangelisches Klinikum Bethel, Remterweg 69-71, 33617 Bielefeld, Germany; 2 Department Sport & Gesundheit, Universität Paderborn, Paderborn, Germany

**Keywords:** Unipolar depressive disorder, Sport and exercise therapy, Outpatient treatment, Recommendations, Evaluation study, Unipolare depressive Störung, Sport- und Bewegungstherapie, Tagesklinische Behandlung, Handlungsempfehlungen, Evaluationsstudie

## Abstract

**Objective:**

The positive effect of sport and exercise interventions on the treatment of unipolar depressive disorder (UDD) is well documented with respect to aerobic exercise. However, few studies have determined the effectiveness of other types of interventions (e.g., weight training, body and mind oriented, qigong or progressive muscle relaxation). Additionally, the effectiveness of specific combined sport and exercise approaches has rarely been investigated. Therefore, recommendations for the use of sport and exercise therapy to treat UDD have been developed.

**Methods:**

This quasi-randomised study used a pretest/posttest design to compare the effectiveness of two different interventions (aerobic activity training vs. combined physical activity training) on psychiatric outcome parameters in a day clinic psychiatric setting. A total of 62 participants were quasi-randomised to one of the two conditions. Affective, cognitive, psychosocial and neuropsychological changes were assessed by a battery of questionnaires before (t1) and after (t2) treatment. Accelerometers were used to assess energy consumption.

**Results:**

The results show that both training interventions have similar effects on the treatment of UDD.

**Conclusion:**

These findings highlight the effectiveness of different physical activities in the treatment of UDD and provide further information for good clinical practice.

## Abstract (de)

**Hintergrund:**

Der positive Effekt von Sport- und Bewegungsinterventionen bei depressiven Erkrankungen ist für den aeroben Ausdauerbereich gut dokumentiert, während andere Interventionen (z. B. Krafttraining, Übungen zur Körperwahrnehmung, Qigong, progressive Muskelentspannung) nur vereinzelt untersucht wurden. Ergänzend wurden kombinierte Trainingsprogramme nur ansatzweise erforscht. Aus diesem Grund beschreiben die Autoren Handlungsempfehlungen für die Sport- und Bewegungstherapie mit depressiven Patient*innen.

**Methode:**

Es handelt sich um eine quasi-randomisierte Evaluationsstudie im Prä‑/Posttest-Design, wobei die Effekte von zwei unterschiedlich akzentuierten Sport- und Bewegungsinterventionen (aerobes Ausdauertraining vs. kombiniertes Trainingsprogramm) auf psychiatrische Parameter im tagesklinischen Setting untersucht wurden. Es nahmen 62 Patient*innen an der Studie teil, die den beiden Gruppen quasi-randomisiert zugeteilt wurden. Mit Hilfe einer Fragebogenbatterie wurden vor (t1) und nach (t2) der Behandlung affektive, kognitive, psychosoziale und neuropsychologische Veränderungen gemessen. Ergänzend wurden Akzelerometer eingesetzt, um den Kcal-Verbrauch zu bestimmen.

**Ergebnisse:**

Die Ergebnisse zeigen vergleichbare Effekte für beide Interventionen auf die psychiatrischen Parameter.

**Schlussfolgerung:**

Die Ergebnisse verdeutlichen den positiven Effekt unterschiedlicher Sport- und Bewegungsinterventionen auf depressive Erkrankungen und liefern wichtige Informationen für die klinische Praxis.

## Introduction

Depressive disorders are a problem for public health care systems worldwide [1], with a lifetime prevalence rate ranging from 15 to 20% [2]. Therefore, clinicians and researchers have become increasingly interested in therapeutic interventions that go beyond established first-line psychotherapy and pharmacotherapy. One option is sport and exercise therapy [3–6]. Its effectiveness has been confirmed in several meta-analyses, thus supporting the application of sport and exercise programs in clinical settings [7–10]. While the general effectiveness of sport and exercise therapy has been well documented, it is still unclear which kind of physical activity should be applied. Domestically, aerobic exercise has been the most commonly used physical activity in study protocols (e.g., [8, 11, 12]). Additionally, the current literature [13, 14] shows that weight training and body and mind-oriented interventions (e.g., qigong, yoga or progressive muscle relaxation) also have positive effects on depression and are often used in clinical settings. Imboden et al. [15] conducted a recent review of 55 meta-analyses and found that aerobic and/or resistance training as well as mind–body exercises are highly effective. Nevertheless, the effect diminished when examining only clinical trials with high-quality standards, and there is little evidence indicating the effectiveness of combined exercise interventions (e.g., a combination of aerobic exercise, body and mind-oriented interventions, and weight training; e.g., [7, 16, 17]). In addition, there is still a lack of guidelines for the use of sport and exercise therapies in the treatment of unipolar depressive disorder (UDD); thus, such interventions are often based on intuition and best-practice models.

Therefore, Weigelt and colleagues [3, 4] provided recommendations for sport and exercise therapy that transform psychotherapeutic measures into exercise programs. The recommendations indicate that combined sport and exercise therapy should 1) promote the spontaneous initiation of actions and cognitive flexibility, 2) allow for mistakes and make them part of the learning process, 3) improve body awareness and the sensation of bodily states, 4) build up physical activity as a positive experience and 5) improve stress coping skills and self-efficacy. Based on these recommendations [3, 4], the present study compares the effects of aerobic activity training and combined physical activity training as part of a complex treatment program for UDD in a day clinic psychiatric setting. In accordance with the current literature, we implemented a combination of 1) aerobic exercise, 2) body and mind-oriented interventions (qigong and progressive muscle relaxation), and 3) general exercise therapy (e.g., strength training). The rationale for examining these two kinds of interventions was as follows: 1) multimodal exercise programs have a positive influence on the health and wellbeing (as signified by physical, functional and quality of life variables) of older adults [17] and 2) combined exercise trainings are already in use at day care clinics, but empirical evidence for such practices is rare [3, 4].

Herein, we investigate whether combined physical activity training confers stronger exercise treatment effects than aerobic activity training. Exercise treatment effects were assessed by a variety of questionnaires. The major hypothesis is that combined physical activity training is more effective than unimodal aerobic activity training with respect to depression severity (main outcome parameter). On an explorative level, we assumed that combined physical activity training would be more effective than unimodal aerobic activity training at improving overall psychopathological symptom severity, body image, social activity and cognitive complaints (secondary outcomes).

## Materials and methods

### Procedure and participants

**Fig. 1:**
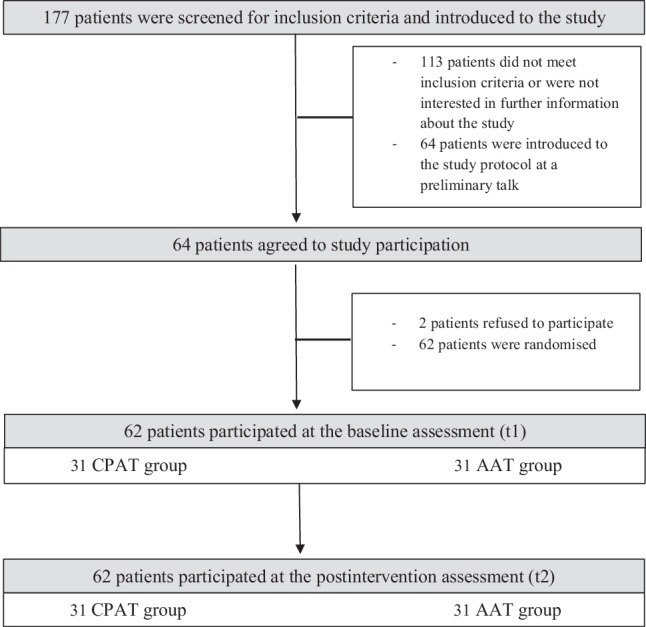
Study flowchart with patients from the combined physical activity training (*CPAT*) group and the aerobic activity training (*AAT*) group

The study was performed at a day clinic located in the city centre. Patients were recruited between 01 June 2013 and 31 November 2014. After admission, clinical psychologists and psychiatrists consecutively screened the patients for eligibility. A total of *n* = 62 participants fulfilled the inclusion criteria and were quasi-randomised to one of the two conditions, i.e., they were alternately assigned to the combined physical activity training (CPAT) group or the aerobic activity training (AAT) group. It was not possible to include a control group without any intervention due to ethical guidelines. Two patients refused to participate before randomisation (Fig. 1). Inclusion criteria were as follows: i) age 18 to 65 years; ii) admission to the day clinic because of a current depressive episode (ICD-10 F32 or F33), dysthymia (F34.1), or mixed anxiety and depression (F41.2); and iii) written informed consent. Patients showing orthopaedic, cardiovascular, and/or internal medicine problems or acute psychosis who were prohibited participation in regular sport and exercise training were excluded. Before the first training session (t1), participants completed the whole battery of questionnaires in a pretest within approximately 1h. The posttreatment assessment (t2) took place when they left the clinic. The study was approved by the ethics committee of the University Münster (date of approval: 15 October 2013) and registered at the German Register of Clinical Trials (DRK; registration number: DRKS00028254). Standards of good clinical practice and the demands of the Declaration of Helsinki were fulfilled.

### Interventions

Training in the combined physical activity training group consisted of a combined sport and exercise training including 60 min Nordic walking, 30 min progressive muscle relaxation, 45 min general exercise therapy and 45 min qigong (four sessions per week). Participants randomised to the aerobic activity training group completed three sessions of Nordic walking (each 60 min) per week. Each session started with a 10-minute warmup, followed by 45 min of Nordic walking and a cool-down phase of 5 min. Both trainings were offered during the whole day-hospital stay for each participant (6 to 8 weeks). Details of the intervention program (combined physical activity training group) and assignment to the recommendations are shown in Table 1. Training intensity was not predefined but measured as energy consumption during each training session. The mean attendance rate for sports and exercise therapy between admission and discharge from the clinic did not differ between the two groups (combined physical activity training group: 4.3 weeks ± 1.82, aerobic activity training group: 4.6 weeks ± 2.0). The usual treatment regimen consisted of extensive visits, pharmacotherapy if indicated, cognitive behavioural therapy, training of social competencies, occupational therapy and weekly sessions with the primary nurse in both groups.

**Table 1:** Details of the intervention program (combined physical activity training group) and assignment to the recommendations by Weigelt and Berwinkel [3] and Weigelt et al. [4]

Intervention	Time/frequency	Content	Recommendation
Nordic walking	1 ×/week and 60 min	Each session started with 10-minute warmup, followed by 45 min of Nordic walking. Training ends with a cool-down phase of 5 min	(4) and (5)
Progressive muscle relaxation	1 ×/week and 30 min	Progressive muscle relaxation with regard to Jacobsen in sitting position	(3)
Qigong	1 ×/week and 45 min	Each session started with a 10-minute warmup, followed by specific qigong exercises. Training ends with a short feedback round	(3) and (2)
General exercise therapy	1 ×/week and 45 min	General exercise therapy included cooperative exercises as well as body awareness training, sports games, strength training, dancing and teambuilding exercises	(1) and (2)

*(1)* promote spontaneous initiation of action and cognitive flexibility, *(2)* allow for mistakes and make them part of the learning process, *(3)* improve body awareness and the sensation of bodily states, *(4)* build up physical activity as a positive experience, *(5)* improve stress coping skills and self-efficacy

### Psychological diagnostics

The following battery of questionnaires was used for psychological diagnostics: 1) the Beck Depression Inventory (BDI II; [18]), a 21-item clinical questionnaire that can be used to evaluate the severity of depression; 2) the Symptom Checklist (SCL-K‑9; [19]) was used to measure the global severity index on a four-point Likert scale with 9 items; 3) the Body Image Questionnaire (QBI-20; [20]) provides information about two independent dimensions of body image, each consisting of 10 items (negative body image [NBI] and vital body dynamic [VBD]); 4) the Social Activity Self-Assessment Scale (SASS; [21]) tests social functions and their impairments with 20 items on a four-point Likert scale; 5) the Multidimensional Self-Esteem Scale (MSES; [22]) consists of 32 items to measure different scales of self-esteem, the two main scales are the general self-esteem scale (GSE) and body self-esteem scale (BSE); and 6) the Subjective Evaluation of Mental Performance questionnaire (Flei; [23]) is a mental performance test of attention, memory, and cognitive flexibility consisting of 35 items on a four-point Likert scale.

### Physiological diagnostics (activity sensors)

During each session, energy consumption was measured as a physiological parameter by using activity sensors (accelerometer Move II, Movisens GmbH, Karlsruhe, Germany). Move II consists of a triaxial acceleration sensor with a range of ± 8 g, a 64-Hz sampling frequency and a 12-bit resolution. The activity sensors were given to each participant before each session and were worn on the right side of the hip.

### Statistical analysis

Potential differences in baseline characteristics between the two groups were analysed using *t*-tests and χ^2^ analysis. All treatment effects were analysed using the intent-to-treat principle [24]. All data were analysed using SPSS 17 (IBM Corp., Armonk, NY, USA). Data from the activity sensors were analysed using the Data Analyser, Sensor Manager and Unisens Viewer software (movisens GmbH, Karlsruhe). A series of analyses of variance (ANOVAs) with repeated measurements were used for the within-subject factor time (t1 vs. t2), the between-subject factor (aerobic exercise training vs. combined sport and exercise training) and their interactions. T‑tests were used for adjusted post hoc analyses (sequential procedure: [25]). Moreover, standardised effect sizes were calculated using Cohen’s *d* [26].

## Results

The characteristics of both groups are presented in Table 2. The statistical analysis (*t*-/χ^2^ test) showed no significant differences (*p* > 0.05) between the groups at baseline with regard to personal data and the mean values of the measurement instruments (Table 2). With regard to average energy consumption, this was 6.19 (± 2.1) kcal/kg and week in the combined physical activity training group and 11.67 (± 2.7) kcal/kg and week in the aerobic activity training group; this difference was statistically significant (*t* (60) = −9.019, *p* < 0.001). The pre- and posttreatment scores of all questionnaires are presented in Table 3.

**Table 2:** Descriptive group characteristics for the combined physical activity training (*CPAT*) group and the aerobic activity training (*AAT*) group

Characteristic	CPAT group (*n* = 31)		ATT group (*n* = 31)		*t/χ*^2^	*df*	*p*-value
*Age in years, mean (SD)*	42.8 (11.89)		41.0 (11.52)		0.60	60	0.551
*Sex, n (%)*					1.13	1	0.288
♂	13	41.9	9	29.0			
♀	18	58.1	22	71.0			
*Diagnosis, n (%)*					13.07	9	0.159
Depressive disorder (F 32 & F 33)	30	96.7	29	93.5			
Dysthymia (F 34.1)	1	3.2	1	3.2			
Mixed anxiety and depressive disorder (F 41.2)	0	0	1	3.2			
*Education, n (%)*					3.63	3	0.304
No graduation	1	3.2	0	0			
Lower secondary school	8	25.8	5	16.1			
Secondary school	7	22.6	13	41.9			
High school	15	48.4	13	41.9			
*Duration of current episode, n (%)*					5.54	4	0.236
1–4 weeks	0	0	1	3.2			
1–3 months	4	12.9	8	25.8			
4–6 months	9	29.0	0	0			
7–12 months	9	29.0	12	38.7			
> 12 months	9	29.9	10	32.2			
*Transfer from inpatient stay, n (%)*					0.08	1	0.776
Yes	8	25.8	9	29.0			
No	23	74.2	22	71.0			
*Number of previous psychiatric treatments, n (%)*					3.3	4	0.507
0	8	25.8	14	45.2			
1	13	41.9	10	32.2			
2–4	8	25.8	6	19.4			
5–7	1	3.2	1	3.2			
8 and more	1	3.2	0	0			
*Hospital stay, n (%)*					0.56	4	0.968
1–2 weeks	2	6.5	1	3.2			
2–4 weeks	5	16.1	6	19.4			
4–6 weeks	7	22.6	8	25.8			
6–8 weeks	9	29.0	9	29.0			
> 8 weeks	8	25.8	7	22.6			
*Current pharmacotherapy, n (%)*					0.08	1	0.767
Yes	24	77.4	23	74.2			
No	7	22.6	8	25.8			
*Regular sporting activity before admission, n (%)*					1.13	1	0.288
Yes	22	71.0	18	58.1			
No	9	29.0	13	41.9			
*Frequency of previous sport activities per week, n (%)*					3.09	4	0.542
0	9	29.0	13	41.9			
1–2	9	29.0	5	16.1			
3–4	8	25.8	10	32.2			
5–6	3	9.7	1	3.2			
Every day	2	6.5	2	6.5			
*Mean attendance of sport- and exercise therapy (weeks), mean (SD)*	4.3 (1.82)		4.6 (2.00)		−0.61	60	0.545
*Energy consumption: kcal/week and kg during the program, mean (SD)*	6.19 (2.08)		11.67 (2.67)		−9.02	60	0.000**

*df* degrees of freedom, *t* t test, *χ*^*2*^ Pearson chi-squared test

*p < 0.05, **p < 0.01

**Table 3:** Outcome measures for the combined physical activity training (*CPAT*) group and the aerobic activity training (*AAT*) group before (*t1*) and after (*t2*) treatment

	CPAT group mean (SD)		ATT group mean (SD)		Main effect “time” F, df (*d*)	Main Effect “group” F, df (*d*)	Interaction “time” × “group” F, df (*d*)
	t1	t2	t1	t2			
BDI II	27.4 (9.47)	22.6 (10.29)	25.8 (10.6)	18.1 (8.40)	51.91, 1** (1.86/0.46)	1.74, 1	2.84, 1
SCL‑K-9	2.0 (0.81)	1.7 (0.88)	1.7 (0.71)	1.4 (0.75)	15.42, 1** (1.01/0.20)	2.18, 1	0.011, 1
QBI-20-NBI	29.8 (10.04)	29.2 (10.17)	31.1 (10.98)	30 (11.26)	2.03, 1	0.164, 1	0.178, 1
QBI-20-VBD	24.1 (7.08)	23.8 (8.57)	26.1 (5.87)	28.5 (6.93)	2.76, 1	3.77, 1	4.03, 1* (0.52/0.06)
SASS	33.2 (6.72)	34.1 (6.80)	33.0 (8.04)	35.6 (6.61)	8.28, 1* (0.74/0.12)	0.144, 1	2.07, 1
MSES-GSE	85.4 (25.08)	91.4 (26.36)	82.6 (23.30)	95.4 (26.75)	23.82, 1** (1.26/0.28)	0.007, 1	3.39, 1
MSES-BSE	35.4 (11.76)	38.4 (12.54)	34.2 (13.16)	39.2 (13.88)	18.10, 1** (1.10/0.23)	0.003, 1	1.01, 1
Flei‑A	22.3 (9.21)	21.4 (8.50)	21.4 (8.87)	20.1 (8.05)	8.60, 1	0.001, 1	0.720, 1
Flei‑M	21.7 (8.83)	20.3 (8.35)	21.4 (9.42)	21.2 (8.66)	13.36, 1	14.44, 1	2.18, 1
Flei‑E	20.7 (8.95)	18.7 (8.32)	20.0 (9.37)	18.6 (7.65)	13.10* (0.60/0.08)	144.2, 1	1.17, 1

*df* degrees of freedom, *F* F value, *d* effect size (Cohen’s d), *BDI II* Beck Depression Inventory, *SCL-9‑K* Symptom Checklist, *QBI-20-NBI* Body Image Questionnaire (negative body image scale), *QBI-20-VBD* Body Image Questionnaire (vital body dynamic scale), *SASS* Social Activity Self-Assessment Scale, *MSES-GSE* Multidimensional Self-Esteem Scale (general self-esteem scale), *MSES-BSE* Multidimensional Self-Esteem Scale (body self-esteem scale), *Flei‑A* Subjective Evaluation of Mental Performance (attention scale), *Flei‑M* Subjective Evaluation of Mental Performance (memory scale), *Flei‑E* Subjective Evaluation of Mental Performance (executive function scale)

*p < 0.05, **p < 0.01

### *Beck Depression Inventory* (BDI II; [18])

Decreasing scores from t1 to t2 indicate a lower level of self-reported depressive symptoms after the intervention in both groups. The within-group effect sizes were *d* = 0.49 for the combined physical activity training group and *d* = 0.80 for the aerobic activity training group. ANOVA indicated that the main effect of “time” was significant (*F* (1, 60) = 51.91; *p* = 0.000) but not the “group” (*F* (1, 60) = 1.74; *p* = 0.192) or the interaction of “time × group” (*F* (1, 60) = 2.84; *p* = 0.097).

### *Symptom Checklist *(SCL-K-9; [19])

Decreasing scores from t1 to t2 indicate a lower level of global severity after the intervention in both groups. The within-group effect sizes were *d* = 0.41 for the combined physical activity training group and *d* = 0.35 for the aerobic activity training group. ANOVA indicated that the main effect of “time” was significant (*F* (1, 60) = 15.42; *p* = 0.000) but not the “group” (*F* (1, 60) = 2.18; *p* = 0.145) or the interaction of “time × group” (*F* (1, 60) = 0.011; *p* = 0.918).

### *Body Image Questionnaire *(QBI-20; [20])

Decreasing scores from t1 to t2 indicate a lower level of negative body image (QBI-20-NBI) after the intervention in both groups. ANOVA indicated that the main effect of “time” was not significant (*F* (1, 60) = 2.03; *p* = 0.160), nor was the “group” (*F* (1, 60) = 0.164; *p* = 0.687) or the interaction of “time × group” (*F* (1, 60) = 0.178; *p* = 0.675).

Regarding the vital body dynamic (QBI-20-VBD), increasing scores from t1 to t2 indicate a higher level after the intervention in both groups. ANOVA indicated that the main effect of “time” was not significant (*F* (1, 60) = 2.76; *p* = 0.102) or “group” (*F* (1, 60) = 3.77; *p* = 0.057), but the interaction of “time × group” was (*F* (1, 60) = 4.03; *p* = 0.049), i.e., the factor “time” was mediated by the factor “group”. A post hoc *t*-test for dependent samples was significant between t1 and t2 for the aerobic activity training group (*t* (60) = −2.61; *p* = 0.014) but not for the combined physical activity training group (*t* (60) = 0.244; *p* = 0.809). A post hoc *t*-test of the mean value differences for the two groups between t1 and t2 was significant (*t* (60) = 2.01, *p* = 0.049). Nevertheless, effect sizes were negligible for the combined physical activity training group (*d* = −0.04) and small for the aerobic activity training group (*d* = 0.37).

### *Social Activity Self-Assessment Scale* (SASS; [21])

Increasing scores from t1 to t2 indicate a higher level of social function after the intervention in both groups. The within-group effect sizes were *d* = 0.1 for the combined physical activity training group and *d* = 0.35 for the aerobic activity training group. ANOVA indicated that the main effect of “time” was significant (*F* (1, 60) = 8.28; *p* = 0.006) but not the “group” (*F* (1, 60) = 0.144; *p* = 0.705) or the interaction of “time × group” (F (1, 60) = 2.07; *p* = 0.155).

### *Multidimensional Self-Esteem Scale* (MSES; [22])

Increasing scores from t1 to t2 indicate a higher level of general self-esteem after the intervention in both groups. The within-group effect sizes were *d* = 0.23 for the combined physical activity training group and *d* = 0.51 for the aerobic activity training group. ANOVA indicated that the main effect of “time” was significant (*F* (1, 60) = 23.82; *p* = 0.000) but not the “group” (*F* (1, 60) = 0.007; *p* = 0.936) or the interaction of “time × group” (*F* (1, 60) = 3.39; *p* = 0.070).

Regarding the body self-esteem scale, increasing scores from t1 to t2 indicate a higher level after the intervention in both groups. The within-group effect sizes were *d* = 0.25 for the combined physical activity training group and *d* = 0.37 for the aerobic physical activity training group. ANOVA indicated that the main effect of “time” was significant (*F* (1, 60) = 18.10; *p* = 0.000) but not the “group” (*F* (1, 60) = 0.003; *p* = 0.955) or the interaction of “time × group” (F (1, 60) = 1.01; *p* = 0.317).

### *Subjective Evaluation of Mental Performance* (Flei; [23])

Decreased scores from t1 to t2 on the Flei‑A scale indicate higher attention after the intervention in both groups. ANOVA indicated that the main effect of “time” was not significant (*F* (1, 60) = 8.60; *p* = 0.135), nor was the “group” (*F* (1, 60) = 0.001; *p* = 0.601) or the interaction of “time × group” (*F* (1, 60) = 0.720; *p* = 0.749).

Decreased scores from t1 to t1 on the Flei‑M scale indicate a higher memory score after the intervention in both groups. ANOVA indicated that the main effect of “time” was not significant (*F* (1, 60) = 13.36; *p* = 0.193), nor was the “group” (*F* (1, 60) = 14.44; *p* = 0.876) or the interaction of “time × group” (*F* (1, 60)= 2.18; *p* = 0.331).

Decreased scores from t1 to t1 on the Flei‑E scale indicate a higher executive function after the intervention in both groups. ANOVA indicated that the main effect of “time” was significant (*F* (1, 60) = 13.10; *p* = 0.032) but not the “group” (*F* (1, 60) = 144.24; *p* = 0.826) or the interaction of “time × group” (*F* (1, 60)= 1.17; *p* = 0.764). Low effect sizes in both groups suggest that mental performance is only marginally influenced by the interventions.

## Discussion

The aim of the present intervention study was to compare the effectiveness of aerobic activity training with combined physical activity training in patients with UDD. The combined physical activity training was conducted according to the recommendations for sport and exercise therapy of Weigelt and colleagues [3, 4]. Depression severity (main outcome) improved significantly in both groups. However, we found no evidence that the combined physical activity training was more effective than the aerobic activity training; thus, we rejected the main hypothesis. Aerobic exercise training even had a better effect on the energetic and movement-related aspects of body image. With respect to all other outcomes (general depression severity, overall psychopathological symptom severity, negative body image, social activity, cognitive complaints), both trainings showed comparable positive treatment effects.

Overall, these findings are consistent with several studies suggesting that exercise interventions are effective in the treatment of UDD (e.g., [8, 11, 12]), even for short intervention periods (6–8 weeks), thus highlighting the importance of physical training even during shorter periods of hospital stays. The results also support the assumption that combined physical activity training can generate similar treatment effects to aerobic activity training with respect to depressive symptoms (general depression severity, overall psychopathological symptom severity, negative body image, social activity, cognitive complaints). Enhancing good clinical practice, these findings deliver important implications for sport and exercise therapy. If both trainings have similar effects on depressive symptoms, a combined low-intensity physical activity training might be suitable for patients for whom aerobic activity training is not applicable (e.g., limitations in physical mobility, cardiac problems, side effects of medication or a lack of motivation). In addition, combined physical activity training allows the integration of various elements from psychotherapy into sport and exercise therapy. This matches perfectly with a multiprofessional perspective and a resource-oriented approach in a complex integrated outpatient care model [27]. Therefore, we argue for the use of a combined physical activity training that is embedded in a continuum of behavioural health care measures.

In contrast, we found a higher benefit for the aerobic activity training group on the energetic and movement-related aspects of body image. The energy consumption was significantly higher for the aerobic activity training group than for the combined physical activity training group (6.19 ± 2.1 kcal/kg and week vs. 11.67 ± 2.7 kcal/kg and week). Hence, the positive effect on body image might be based on the higher training intensity. Thus, for patients who are able to engage in aerobic activity training, aerobic activity training might be preferable if body image-related symptoms are relevant.

However, pre-/posttreatment effect sizes in the present study were small compared to previous studies with medium and high effect sizes [8, 28–30]. This may be because we studied less severely ill patients from an outpatient day clinic setting with less potential for symptom improvement, while other studies focused on inpatients. Additionally, as mentioned above, the intervention period of this study was rather short in comparison to other studies that report a median duration of 10 (and up to 16) weeks [8]. To take up the point mentioned above again, the training intensity (combined physical activity training group: 6.19 ± 2.1 kcal/kg and week; aerobic activity training group: 11.67 ± 2.7 kcal/kg and week) may have been too low compared to the 70–80% of the maximum heart rate that has been targeted in other studies (e.g., by jogging or training on a treadmill [30]).

A strength of the present study is the comparison of two different sport and exercise therapeutic measures within a homogeneous day clinic group under realistic conditions of a psychiatric day hospital. However, some limitations of the study should be mentioned as well. Possible differences between the training groups with regard to the improvement in depressive symptoms might have been masked by the parallel use of other therapies in the day clinic. Since it was not possible to also examine a waitlist control group (due to restriction by the ethics committee), we also do not know what proportion of the effect is due to the training programs and not to the other therapies. Additionally, the rather small sample size may have prevented us from revealing differential effects. However, we planned the intervention program based on the recommendations mentioned above under ecologically valid and realistic conditions of a psychiatric day hospital, where the study was integrated into the regular therapeutic routine of the clinic. Under these conditions, we only measured energy consumption as a physiological parameter and were otherwise unable to report or assess detailed training principles (e.g., specificity, progression, overload). Further studies should include more physiological parameters and should consider principles of exercise training for these studies to be precisely reproducible. Finally, we cannot exclude the possibility that the results are biased because full randomisation was not possible. However, patients in both groups were comparable with respect to demographic and clinical variables.

In summary, the present study shows that the effects of a combined sport and exercise program based on the guidelines of Weigelt and colleagues [3, 4] do not differ from those of aerobic activity training with respect to most of the collected parameters. An exception is body image, for which aerobic activity training seems to be more effective. These findings indicate the positive effect of sport and exercise programs in the treatment of UDD.

## Abbreviations

AAT: Aerobic activity training group
BDI II: Beck Depression Inventory
BSE: Body self-esteem scale (MSES)
CPAT: Combined physical activity training group
Flei: Subjective Evaluation of Mental Performance Scale
GSE: General self-esteem scale (MSES)
MSES: Multidimensional Self-Esteem Scale
NBI: Negative body image (QBI-20)
PMR: Progressive muscle relaxation
QBI-20: Body Image Questionnaire
SASS: Social Activity Self-Assessment Scale
SCL-K‑9: Symptom Checklist
UDD: Unipolar depressive disorder
VBD: Vital body dynamic (QBI-20)
